# Physical Therapist and Patient Perspectives on Mobile Technology to Support Home Exercise Prescription for People With Arthritis: A Qualitative Study

**DOI:** 10.7759/cureus.55899

**Published:** 2024-03-10

**Authors:** Nancy M Gell, Paula A Smith, Mariana Wingood

**Affiliations:** 1 Rehabilitation and Movement Science, University of Vermont, Burlington, USA; 2 Implementation Science, Wake Forest University School of Medicine, Winston-Salem, USA

**Keywords:** technology, physical therapy, mobile apps, osteoarthritis, exercise

## Abstract

Introduction

Mobile applications (apps) for exercise prescription may enhance communication between healthcare providers and patients while addressing common barriers to exercise among people with osteoarthritis. However, little is known about preferences and barriers to the use of mobile apps by physical therapists or people aging with osteoarthritis. Therefore, we aimed to examine physical therapist and patient perspectives on mobile apps to support physical therapist-prescribed home exercise for people with osteoarthritis.

Methods

Eighteen physical therapists and 17 individuals with a history of physical therapy for osteoarthritis participated in the study. Focus groups (n = 6, three each with physical therapists and patients) were conducted to gather perspectives on three commercially available mobile apps designed for physical therapy exercise prescription. Semi-structured questions assessed feature preferences, ease of use, exercise completion support, clinical feasibility, and potential barriers and facilitators to using the apps. Recordings of the focus groups were transcribed verbatim. The study team iteratively coded transcripts using thematic analysis.

Results

Perspectives of patients and physical therapists intersected but differed on the mobile apps. All patients preferred video exercise prescription over traditional methods and both patients and therapists predicted challenges and opportunities with mobile communication. Four themes emerged: accountability, data-driven, communication boost, and duality of technology. Facilitators of home exercise through mobile apps included exercise tracking, feedback loop, personalization, and the potential for reduced in-person visits. Barriers included technological challenges, complex interface design, lack of universal applicability, and security concerns.

Conclusion

Mobile app technology has the potential to support exercise adherence for people with osteoarthritis. However, patients’ and therapists’ perspectives differ and highlight numerous challenges that limit the universal clinical adoption of this technology. While physical therapists acknowledged the potential to improve the rehabilitation experience with mobile apps, there was concern about reimbursement and time constraints in the current fee-for-service environment.

## Introduction

Osteoarthritis is the most common cause of disability in the US, affecting approximately 54 million people [1]. Current projections estimate a 49% increase in the prevalence of osteoarthritis with 11.4% of all adults experiencing mobility limitation as a result of osteoarthritis [2]. The mobility limitations of osteoarthritis are a well-defined barrier to physical activity, which complicates the management of other chronic diseases, such as diabetes and cardiovascular disease, and results in higher mortality risk compared to people without osteoarthritis [3]. Given the increasing burden of osteoarthritis at a personal, social, and health system level, strategies are needed to prevent and manage mobility impairments due to osteoarthritis.

In the absence of curative treatment, exercise is a leading option to manage osteoarthritis symptoms [4]. A recent meta-analysis identified exercise therapy as a superior intervention to nonsteroidal anti-inflammatory drugs in managing knee osteoarthritis-related pain [5]. Older adults with osteoarthritis who perform strengthening exercises, also referred to as exercise therapy, demonstrate reduced pain, increased quality of life, and improved overall function [6]. However, barriers to exercise, including a desire for professional guidance, low self-efficacy, fear of injury, and access to exercise opportunities impede the majority of people with osteoarthritis from engaging in exercise and physical activity [7,8].

Physical therapist-directed exercise is an evidence-based treatment and provides the oversight that many people with osteoarthritis desire. However, completion of home exercise, a critical component of physical therapy and the necessary precursor to increasing physical activity is low, with estimates between 39% and 58% [8,9]. Usual care physical therapy treatment includes exercise prescription through paper or virtual handouts with brief instructions and static pictures or stick figure drawings. Such approaches do not incorporate the recommended methods of improving exercise completion, including enhancing behavioral capability, fostering self-efficacy, and engaging social support [10].

Technology products such as mobile applications (apps) have become widespread tools for chronic disease management [11]. Mobile apps can support the communication between physical therapists and patients while addressing common barriers to exercise among those with osteoarthritis using options such as reminder systems, tracking options, video demonstrations of exercises (by the physical therapist demonstrating, a pre-made library of videos, or videos of the patients themselves doing their exercises), and means to communicate between in-person sessions. A pilot study of older adult females with knee osteoarthritis demonstrated greater exercise completion and reduced knee pain in the group who used an app with their physical therapy and home exercise program compared to those who did not use an app [12]. Yet, little is known about preferences and barriers to the use of mobile apps by physical therapists or people aging with osteoarthritis. Therefore, the purpose of this study was to examine patient and physical therapy provider perspectives on mobile apps to support prescribed home exercise for the management of osteoarthritis.

## Materials and methods

Study design

We conducted a qualitative study to describe both patients’ and physical therapists’ experience of using apps within physical therapy and to explore the usability, functionality, and design of available features within exercise prescription apps. We also collected quantitative data to better describe our study participants, including their experience with different exercise prescription modes, individual experiences with tracking exercise completion, and physical therapists’ methods used to communicate with patients. All procedures were approved by the Institutional Review Board and all participants provided informed consent before beginning the study.

Participants

Participants were recruited through clinic invitations and community postings. We recruited people 50 years and older who had previously received physical therapy for the treatment of osteoarthritis (patients), and we recruited physical therapists with at least one year of clinical experience working with people with osteoarthritis in an outpatient setting.

Procedures and measures

Quantitative Data

After obtaining consent, participants with osteoarthritis completed a demographic and health history questionnaire. Data collected included age, sex, years since osteoarthritis diagnosis, treatment used to manage osteoarthritis symptoms, time since last physical therapy episode of care for osteoarthritis, mode of exercise prescription from the physical therapy, and experience with tracking exercise participation.

Physical therapist participants completed a demographic and work history questionnaire after consent. Data collected included age, sex, years of licensure, average number of patients per month treated for osteoarthritis, methods used to provide exercise prescriptions, methods used to communicate with patients, and experience with tracking exercise participation with patients.

Qualitative Data

We conducted six focus groups: three groups included patients and the other three groups consisted of physical therapists. The focus groups were moderated by a master’s level senior research associate with prior training and experience conducting interviews and focus groups. The moderator used a semi-structured discussion guide. The discussion guide provided the focus group moderator with questions, probes, and cues [13]. The guide had the following format: (1) an opening to promote conversation; (2) an introduction of the topic; (3) a transition to key questions; (4) key questions related to the study purpose; and (5) ending question that brings closure [13]. All discussions were recorded. A research assistant took accompanying field notes. The recordings were transcribed verbatim.

For all focus groups, the opening conversation was a discussion about their experience with the exercise prescription aspect of physical therapy for the treatment of osteoarthritis. The familiarization stage started with a discussion of exercise prescription apps in general. Then, each participant received an iPad (Apple Inc., Cupertino, CA) pre-loaded with three mobile apps designed for exercise prescription in the physical therapy setting. The mobile apps selected had similar and unique features compared to the other apps. Similarities across the three apps were free access for patient use but fee-based access for the prescribing physical therapist, data sharing and communications options between patient and physical therapists, and inclusion of a reminder system (pop-ups, sounds, or messages). Unique features of App #1 included the following: (1) exercise prescription through a video of the patient doing the exercises themselves; (2) an interface that allowed exercise completion tracking; (3) difficulty and pain rating for each exercise; (4) an option to connect with wearable physical activity tracking devices; and (5) use of standardized questionnaires for outcomes data collection. Unique features of App #2 included the following: (1) the physical therapist has the option of prescribing exercises through pre-recorded videos, customized patient videos, or static photos e-mailed via PDF document; (2) communications via text or audio messages; and (3) offline accessibility. Unique features of App #3 included the following: (1) the physical therapists can choose exercises from a library of pre-recorded videos, photos, or drawings of exercises; (2) availability of standard protocols; and (3) offline access.

Participants were led through a demonstration of each mobile app and then provided time to explore and test the app for themselves. During the transition phase of the focus group, participants were instructed to consider three features of the apps as they used them: ease of use (i.e., usability), functionality, and interface design. After testing the mobile apps, participants were asked to discuss the usability, functionality, and design of each interface. The focus group concluded with a discussion of options for communication outside of in-person visits, preferred features of a mobile app for exercise prescription, and interest in using an app for exercise prescription.

Data analysis

Descriptive statistics (e.g., means, standard deviations, and frequencies) were calculated from responses to the brief questionnaires using STATA SE 16 (StataCorp LLC, College Station, TX). Data across the six focus groups were aggregated for analyses to obtain divergent and varied perceptions on factors that may impact the use of mobile home exercise prescription apps. All transcripts were read by three independent researchers using an inductive approach. Each member of the research team independently coded four transcripts. They then met to review assigned codes and discussed agreement and differences in the codes generated. The researchers then completed a second round of coding using the agreed-upon codes. Subsequently, the researchers categorized the codes under four themes and checked the themes against the transcripts. An agreed-upon summary matrix for each theme was outlined, including subthemes and quotes. The authors also generated a list of key barriers and facilitators, articulated by participants, to the adoption of mobile apps in physical therapy practice by patients and physical therapists.

Qualitative standards for rigor

Ensuring rigor in qualitative research means assessing the study’s dependability, credibility, transferability, consistency, and confirmability [14]. Methods used to ensure rigor included the following: (1) using a question guide to run the focus group; (2) recording the interviews and transcribing them word by word; (3) maintaining a clear trail of data by creating tables for each step of the coding process; (4) in-depth description of the qualitative themes; (5) using self-reflective strategies such as debriefing with the research team; and (6) having multiple coders [14,15]. To further enhance the rigor of the study, we followed the Standards for Reporting Qualitative Research.

## Results

Participants were 18 physical therapists (83% female; mean clinical experience = 16 ± 10.8 years) and 17 patients (77% female; mean age = 64.0 ± 6.8 years). See Table 1 for details. The most frequent methods used for exercise prescription reported by physical therapists were printed handouts (n = 14, 82%). None of the patients reported receiving their exercise prescription using a video platform but 39% (n = 7) of the physical therapists reported using video occasionally or sometimes. One of the patients and none of the physical therapists had experience with a mobile app for exercise prescription during a physical therapy episode of care.

**Table 1:** Participant characteristics

	Patient participants	Physical therapist participants
	n = 17	n = 18
	Mean (SD) or n (%)	Mean (SD) or n (%)
Age (years)	64 (6.8)	41.9 (11.3)
Female	13 (77%)	16 (89%)
Years licensed		16.1 (10.8)
Number of patients per month treated for osteoarthritis		15.9 (17.7)
Years since the osteoarthritis diagnosis		
0-1	0	
2-3	1 (6%)	
4-5	0	
5-9	8 (50%)	
>10	7 (35%)	
Months since the last physical therapy episode of care	8 (7.6)	
Osteoarthritis location		
Knee	9 (56%)	
Hand	8 (47%)	
Arm	2 (13%)	
Back	7 (44%)	
Ankle/foot	2 (13%)	
Hip	7 (44%)	
Shoulder	2 (13%)	
Neck	6 (38%)	
Osteoarthritis treatments currently using		
Medication	14 (88%)	
Acupuncture	1 (6%)	
Massage	1 (6%)	
Heat	1 (6%)	
Ice	1 (6%)	
Exercise prescription mode		
Hand-written instructions	12 (71%)	15 (83%)
Hand drawn pictures	12 (71%)	14 (78%)
Exercise prescription software printouts	8 (47%)	18 (100%)
Demonstration only	2 (12%)	0
Photos	1 (6%)	11 (61%)
Mobile application	1 (6%)	0
Phone/device videos	0	7 (39%)
Method for exercise completion tracking		
None	9 (53%)	1 (6%)
Calendar	3 (18%)	3 (7%)
Written checklist	3 (18%)	1 (6%)
Mentally, in head	5 (29%)	Not applicable
Verbal report	Not applicable	15 (83%)
Methods used to communicate with patients outside of clinic visits		
Not applicable (does not communicate outside of in-person visits)		9 (50%)
Email		11 (61%)
Phone call		7 (39%)
Text		3 (7%)

We identified four themes: (1) accountability through reminders and trackers; (2) data-driven; (3) communication boost; and (4) duality of technology. Additionally, the patients and physical therapists noted unique facilitators and barriers with mobile communication and app support for exercise compared to paper handouts for exercise prescription.

Accountability through reminders and trackers

There was consensus among patients and physical therapists that features embedded in the mobile apps enhance accountability for home exercise program completion. Highlighted features of apps that enhance accountability include daily pop-up reminders to complete the exercise, the ability to record when and how often exercises were completed, and options to convey symptoms and level of difficulty associated with individual exercises. In discussing the reminder features, patients reported their intentions of performing their exercises, but then getting distracted or engaged in other activities. The reminders serve as a means to redirect focus to their exercise intentions: “Oh gee, I need to do that” (Patient 3). Similarly, the physical therapists noted that patients often verbalize forgetting to do their exercises; therefore, the pop-ups, along the lines of “It's time to do your exercises? Or have you done your exercises yet?” (Physical Therapist 4), would be useful to increase accountability.

The ability to record exercise completion was also noted as a means of increasing accountability. Patients verbalized that it would help them see progress toward completing the recommended exercises. For example, Patient 1 said, “…it would keep me motivated and keep me on track, especially the one with the verification counter stating the fact that all was accomplished.” Patients also discussed how exercise completion tracking can foster accountability as an active participant in physical therapy. “If you only did half, you say to yourself ‘You know, I’m going for therapy, and I see that I’ve only done half of what I should have.’ And, you know, I’m responsible for why I’m not moving along a little better” (Patient 15).

Another source of accountability was the knowledge that their physical therapists would see their exercise completion. As Patient 11 stated, “I have a feeling if I know my PT knows that I’ve not been compliant, I’d be a lot more compliant.” Similarly, Physical Therapist 2 stated, “If they know that I’m seeing that, then there will probably be a little more accountability, versus "ok see you next week.”

Further, physical therapists reported that the accountability enabled through the app would provide them with the needed time to focus on in-person conversations on exercise-related challenges and improvements, rather than completion success. For example, Physical Therapist 12 states, “I don't think I take the time with my system now to always dive into those issues.” While Physical Therapist 11 stated, “I don’t take the time to dive into patient feedback on exercise compliance, and it’s nice to have those features automatically pop up.”

Data-driven

The data provided through the mobile apps were considered an advantage among patients and physical therapists. Both the patient and physical therapy participants found that data-sharing about exercise completion, ease/difficulty of exercises, reasons for not completing the exercises (e.g., pain, difficulty, symptoms), and the progression of exercise completion is beneficial. Patients felt that data conveyed through a mobile app would benefit patients in seeing progress reports (e.g., number of exercises completed, number of days each week the exercises were completed, and completed exercises as a percentage of those assigned). Patients verbalized that the ability to provide input about ease of exercises or pain while doing the exercises provides the physical therapist with contextual feedback that could then inform treatment changes or progression. This is highlighted by Patient 7, “If I could say ‘this is almost too easy,’ then the therapist could prescribe different exercises by updating the app.” Similarly, Patient 8 noted, “It may be important for that physical therapist to know that maybe, at that point, something else is going on or that's too much exercise, it gives the therapist feedback” and “because if I’m only able to complete a few of them and rate my pain as really high, if may be important for that physical therapist to know.”

For physical therapists, the data provide insight into the potential need to modify or progress the exercises. For example, seeing that most of the exercises were marked as “easy” was a clear indicator that the patient was ready for progression. Conversely, having data regarding which exercises resulted in increased pain would prompt additional diagnostic testing or exercise modification. Physical therapists also appreciated receiving data through the app that they may not have time to discuss during treatment. For example, Physical Therapist 7 stated, “I guess I always really want to know if there are certain ones or one that was too hard” and Physical Therapist 12 added that they would appreciate additional communication about “how they were feeling that day, what their pain level was during the exercises, [and] how difficult it felt for them to do it.”

Additionally, data collected outside of physical therapy would be valuable for tracking nutrition, pain monitoring, and identification of activities that may aggravate or relieve pain. For example, Physical Therapist 18 stated “Lately I've been also having people try to track their activity and their diet on those days they are also having more pain. And this is a really nice way because people will come in with pieces of paper like this where they have thousands of notes and they're like I don't remember which day, so it's kind of nice that they can also put those comments right in at the time they do their exercises.”

Communication boost

The opportunity to communicate through a mobile app between in-person visits was perceived differently by the patients and the physical therapists. Patients perceived the “chat” feature embedded in one of the apps as a potential means to enhance communication between in-person visits. They identified circumstances where communication through the app, on topics such as completing the exercises, feedback on results from the exercises, and progression of the exercise program prior to the next in-person visit, were all advantages of maintaining communication through the app and increasing accountability. Interestingly, both patients and physical therapists expressed concern about the potential expectation of instant communication and that the physical therapists would be challenged to respond to chat messages in a timely manner. Patient 4 also noted that the benefits “would depend on how often you’re seeing the therapist. If you’re only going once a week, I would want some communication in between.”

Concerns voiced by physical therapists about keeping up with messages included a lack of time and reimbursement for time spent responding to messages, and needing to be accessible to patients outside of appointments. These concerns were emphasized by Physical Therapist 16 who stated, “It could be time consuming…if you're seeing in the course of a week what, 40 patients maybe, and they all…some a couple [or] three times a week so let's say 20. You know you're getting 20 emails every day and even if you're only saying yup, nope, yup it's still gets time-consuming.” Physical Therapist 18 echoed these feelings by stating, “This is all time consuming.” Further, Physical Therapists 12 and 7, respectively, stated “I think the chat feature is too much access” and “You don’t want to lose rapport with patients, its about relationships. I think the chat feature is too much access.”

Another potential benefit of the chat feature identified by some participants was the ability to enhance the interactions between providers and patients who had difficulty participating in in-person therapy, for example, those with caregiver responsibilities or transportation challenges. “I think it's very important, transportation issues and the ruralness of our state this kind of app could be used in lieu of people in like snowstorms who are supposed to go to PT” (Patient 16). However, there was little interest in reducing or eliminating in-person visits secondary to app communication. In other words, the chat feature was perceived as beneficial for optimizing communication between visits, but not as a replacement for in-person visits unless necessary due to circumstances such as weather or transportation barriers.

Another communication component discussed was related to the delivery mode. Physical therapists thought that apps with a feature for therapists to create their own videos allowed them to communicate tailored verbal cues for correct postures or movements, a feature not available in a paper format or in apps that use a library of pre-made videos only. For example, Physical Therapist 3 verbalized that the disadvantage of paper is that “it’s not really showing the movement of the exercise.” Physical Therapist 1 further emphasized the benefit of using applications over paper drawings by stating the following “I went to PT school, not art school as I tell many of my patients, so many of the stick figures don't always do justice for the patients.” Furthermore, physical therapists verbalized that having the ability to provide visual and auditory cues ensures that the home exercise program is available for different learning styles. This was emphasized by the following statement, “What's nice about all of these is having a visual for people who are visual learners. But then also people who are oral learners, have got the narration to go along. So each of those learning styles is addressed” (Physical Therapist 7).

Duality of technology

Both patients and physical therapists noted specific advantages to a mobile app for exercise prescription, including ease of access, the ability to hear their physical therapist provide instructions on a video, video over drawings or printouts, easy management of updating the exercise program, and communication features as noted previously. There was also a clear acknowledgment of barriers that often accompany the adoption of new technology that could hinder the therapeutic relationship and result in frustration and less focus on direct patient treatment and education.

One example of duality was related to the reminder feature previously mentioned. In general, patients felt that reminders are helpful. For example, Patient 4 stated, “I have an app on my phone that it’s a glorified pedometer, and it will remind me that I haven’t done all my walking today, and that is helpful.” However, the patients also felt that the reminders have the potential to be overwhelming and annoying. Therefore, they suggested the need for a “reminders off” option.

Related to incorporating technology in clinical practice, physical therapists were concerned about the need to use multiple devices in the clinic for app usage. Physical Therapist 9 stated, “One thing that would make it tough for us is that we have laptops but we all don’t have tablets or phones, I mean we do have phones but once in a while I would like to not have my personal phone in use. So, to have my laptop and an iPad mini to juggle would be tough.” Physical Therapist 11 noted, “I feel like we already are required to spend a lot of time on our laptops anyways, and I don’t really like that, so I feel like adding another thing to it will always make me feel like I’m on my computer and not talking.” The patients were more optimistic about the advantages of technology as part of their treatment, “I really like the idea of the apps and having them available whenever you want, and turning them on wherever you are and not losing the papers, or throwing them out, or what have you” (Patient 16). However, both groups acknowledged that common challenges with new technology adoption could result in frustration and potentially take time away from the focus on patient treatment and education.

Barriers and facilitators

Throughout the focus group discussion, and, in particular, during the hands-on sessions with the three mobile apps for exercise prescription, participants noted barriers and facilitators to adopting mobile health. A summary of the most commonly cited barriers and facilitators is presented in Table 2. Despite the barriers to using mobile technology, all of the patient participants stated they preferred to receive their exercise prescription through a mobile app, with the video feature being the most preferred aspect of the technology. Among the physical therapist participants, there was an acknowledgment of the potential value of incorporating mobile apps for exercise prescription, but 50% indicated they preferred to continue using paper handouts.

**Table 2:** Barriers and facilitators to mobile app use for exercise prescription

Barriers	Facilitators
Technological challenges	Exercise tracking
Complex interface design	Feedback loop
Lack of universal applicability	Reassurance
Security concerns	Potential for reduced in-person visits
Learning curve for names of exercises within an app and navigating a new app	Improved clarity

## Discussion

This study provides insight into the use of mobile apps in exercise prescription from the perspectives of physical therapists and patients with osteoarthritis. Key findings demonstrate that mobile apps have features with the potential to extend traditional exercise prescription through the constructs of supportive relationships, efficient information exchange, and shared action planning, as described previously by Jesus and Silva [16]. Physical therapists and patients were in alignment on the potential for app use to improve accountability and enhance the therapeutic relationship. Although both groups acknowledged the likelihood of technology challenges when using apps for exercise prescription, patients perceived the benefits as outweighing the challenges whereas the physical therapists expressed more concern about challenges disrupting rather than enhancing communication.

The benefits of using mobile applications within health care include data collection, therapeutic and observational gamification, patient empowerment, and increased communication [17]. The data collection related to the completion of the exercises and symptoms experienced during the exercises was a benefit voiced by patients and physical therapists. A systematic review examining smartphone applications within physical rehabilitation highlighted the value of using phones to monitor activity completion and symptoms, as well as provide a platform for communication. Within the systematic review, they also discussed the potential of using the smartphone as a body-worn sensor that can provide insight into the patient's movements [18]. Our study participants also voiced that this type of tracking may increase accountability and thus foster exercise completion. These findings are highlighted by a meta-analysis examining the impact of activity tracking on increasing physical activity among adults [19]. Within the meta-analysis, they found that the use of mobile activity tracking has a positive effect on overall physical activity, corresponding to an increase of 1850 steps per day, and highlighted that interventions that include text-messaging and personalization features were significantly more effective in increasing daily step counts [19].

Improved communication was another benefit voiced by our participants. This finding has been emphasized in multiple studies, particularly those conducted in rural settings and among patients with limited mobility [20-22]. A systematic review examining available mobile health apps for managing acute or chronic pain among older adults identified that mobile apps provided both formal and informal communication opportunities, such as chat features, tracking symptoms and activities, and providing support and coaching [23]. A pilot study, targeting older adults with osteoarthritis and aimed at using a mobile app to manage osteoarthritis pain, identified a positive increase in communication [24]. Similarly, Eustache et al. (2022) identified that using a mobile phone app that provides educational material, symptom tracking, and a chat function for patients recovering from post-colorectal surgery led to increased communication and subsequently decreased health complications and healthcare utilization [25].

Despite the multiple benefits of using mobile applications and their widespread availability, the overall adoption of mobile apps within health care is limited with multiple challenges cited as the rationale. These challenges include lack of app quality, lack of evidence of clinical effectiveness, lack of integration between app platforms and information technology systems, usability, access, reimbursement, concerns about security, privacy, and financial and time burden for patients and organizations [26]. The challenges related to lack of reimbursement, time, and access were highlighted in the current study. These challenges relate directly to an overarching concern about increasing burnout risk voiced by the physical therapist participants [27]. Thus, it is essential that these challenges are addressed prior to increasing the adoption of mobile applications within clinical practice.

There was a consensus among patients with osteoarthritis for exercise prescription preference through mobile apps over paper handouts. These findings are further highlighted by a participant preference trial of two self-managed fall prevention exercise interventions that compared digital programs to paper booklets. Within this study, the older adult participants who received the digital program had a lower attrition rate, exercised a significantly greater number of minutes per week, and had a greater percentage of participants who continued exercising 12 months post-intervention [28]. Similarly, a single-blind randomized controlled trial identified that a greater percent of participants who received a mobile video-guided home exercise program performed their exercises three months into the start of the study, had higher self-efficacy for exercise, and made significantly greater functional gains than those who received a paper-based home exercise program [29]. These differences found in other studies provide context to our participants' preferences for mobile apps over paper handouts and highlight the advantages of using mobile apps or technology-based home exercise programs that incorporate videos.

We acknowledge a number of limitations with the current study. None of the participants had prior experience with using mobile apps for exercise prescriptions. Participants were able to convey interests and concerns about the app features, but these were based on a relatively brief examination of three different apps and not from extended experience with using the apps. Given their willingness to participate in discussions about mobile apps, the participants may be more inclined to consider their use and are not representative of all patients with osteoarthritis. We intentionally chose three apps with features unique to each other by comparison, but the apps by no means were inclusive of all potential app options that may influence decisions to use in clinical practice. Finally, we acknowledge the technology gap among older adults. While rates of mobile technology adoption are increasing, they are still lowest among older adults compared to other age groups. However, technology use is higher among older adults with disabilities [30], which illustrates the potential of using technology within rehabilitative care for older adults.

## Conclusions

Mobile app technology has the potential to support exercise completion for people with osteoarthritis. However, patients’ and therapists’ perspectives differ and highlight numerous challenges that limit the universal clinical adoption of this technology. Although the use of mobile apps for exercise prescription is not feasible for all patients or clinical situations, the current findings suggest patients desire more technology integration in their physical therapy treatment that therapists may not be aware of or willing to consider. Future research is needed to demonstrate how the incorporation of mobile apps in physical therapy treatment impacts patient outcomes.
